# Patient Safety in Medical Education: Students’ Perceptions, Knowledge and Attitudes

**DOI:** 10.1371/journal.pone.0135610

**Published:** 2015-08-31

**Authors:** Bahram Nabilou, Aram Feizi, Hesam Seyedin

**Affiliations:** 1 Social Determinants of Health Research Center, Urmia University of Medical Sciences, Urmia, Iran; 2 Patient Safety Research Center, Urmia University of Medical Sciences, Urmia, Iran; 3 School of Health Management and Information Sciences, Iran University of Medical Sciecnes, Tehran, Iran; University Health Network and University of Toronto, CANADA

## Abstract

Patient safety is a new and challenging discipline in the Iranian health care industry. Among the challenges for patient safety improvement, education of medical and paramedical students is intimidating. The present study was designed to assess students’ perceptions of patient safety, and their knowledge and attitudes to patient safety education. This cross-sectional analytical study was conducted in 2012 at Urmia University of Medical Sciences, West Azerbaijan province, Iran. 134 students studying medicine, nursing, and midwifery were recruited through census for the study. A questionnaire was used for collecting data, which were then analyzed through SPSS statistical software (version 16.0), using Chi-square test, Spearman correlation coefficient, F and LSD tests. A total of 121 questionnaires were completed, and 50% of the students demonstrated good knowledge about patient safety. The relationships between students’ attitudes to patient safety and years of study, sex and course were significant (0.003, 0.001 and 0.017, respectively). F and LSD tests indicated that regarding the difference between the mean scores of perceptions of patient safety and attitudes to patient safety education, there was a significant difference among medical and nursing/midwifery students. Little knowledge of students regarding patient safety indicates the inefficiency of informal education to fill the gap; therefore, it is recommended to consider patient safety in the curriculums of all medical and paramedical sciences and formulate better policies for patient safety.

## Introduction

Health care services have improved considerably in the light of medical advances; however, these advancements were considered as major threats to health care industry [1]. For example, in 1999 the US Institute of Medicine (IOM) estimated 44000–99000 deaths every year as a result of preventable medical errors [2]. Other studies have indicated adverse events as an international concern [3, 4].

In developed countries, with sufficient funds and modern technology, one out of ten patients is injured [5]. A study estimated ten to eighteen percent of hospitalized patients are injured due to medical error [6]. While, the burden of unsafe care is unclear in developing countries where inappropriate infrastructure, technology and insufficient or even unskillful human resources have caused higher possible risk of harm to the patient in hospitals and in primary care compared with developed countries [5]. Lack of a well-developed system of medical error reporting in these countries has increased the ambiguity. In Iran, there are no official statistics on the incidence of medical errors; however, the results of some studies increase concern regarding the rate of medical errors [7, 8].

Over the past decade, many interventions have been used to address medical errors and improve patient safety, but the prevailing organizational culture in health care environment has been one of the major obstacles. Attitude of physicians to medical errors is one of the most important components of safety culture. A suitable education is suggested as the best strategy to improve appropriate attitude toward patient safety [9]. Some studies suggested the empowerment education for medical and paramedical staff in hospitals and health centers and some others focused on formal educational programs [10] and suggested including patient safety in undergraduate educational programs as a necessity [9]. In some fields, such as medical education this is a necessity [11]. Without a proper educational environment and appropriate training of quality and safety, responding to legitimate demands of the public will be ineffective and temporary [12].

Declaration of Helsinki endorsed the role of education: “Education plays a key role in improving patient safety and we entirely support the development, publishing and provision of patient safety education” [13]. The World Health Organization has recently developed a comprehensive patient safety curriculum [14]. Considering these changes in the educational curriculum of students at all educational levels is a challenge and demands appropriate policy and genuine commitment from senior managers [15].

After a health reform in 2009 in Iran and using clinical governance as a model of health care quality improvement, the issue of patient safety was taken into consideration as a key element of the clinical governance [7, 16]. The Iranian Ministry of Health and Medical Education established a clinical governance office in the Ministry to assure implementation of clinical governance in all hospitals and develop a supportive culture for health care quality. Also, the accreditation system of Iran in health care started to bring the issue of patient safety into the consideration [17]. This means that the health system demonstrates its commitment to patient safety. Well-educated human resources and their perceptions and attitudes play crucial roles in this regard [18]. This study aimed to investigate perceptions, knowledge and attitudes of students toward patient safety education. Students' perceptions encompasses causes and management of errors and students’ attitudes include their interest in education and skills related to patient safety.

## Material and Methods

This cross-sectional study was conducted in 2012 in teaching hospitals affiliated with Urmia University of Medical Sciences (UUMS), West Azerbaijan province, Iran. The study population (N = 134) included students with two month experience in the field, all medical interns, nursing and midwifery students taking their clinical training in teaching hospitals. Because of the small number of students and the possibility of their unwillingness to participate in the study, all of the students were included. Questionnaire completion was voluntary and anonymous. Those who volunteered, their verbal consents were obtained. The study was approved by the ethic committee located in deputy of research affairs at UUMS with the reference number of 76 in Nov, 2011.

A two-part questionnaire was used to collect the data. The first part was about demographic information of the students; including age, sex and educational level. The second part consisted of 26 questions: 12 questions asked students' perceptions of patient safety, including two categories of causes of error and error management, 6 questions were about students' knowledge on patient safety, and 8 questions were about students' attitude towards the teaching of patient safety in the university. A five-point Likert scale (1 = strongly disagree/very poor, 2 = disagree/poor, 3 = neutral/fair, 4 = agree/good, 5 = strongly agree/very good) was used to measure the perceptions and attitudes of the students.

The questionnaire was adapted from the studies of Madigosky et al. [19] and Leung [20]. Translation and back-translation was used to develop a Persian version of the questionnaire. To test the validity of the questionnaire it was reviewed by the research team and four experts in patient safety. They were members of the patient safety committee of the University and were involved in running patient safety seminars at the University with nursing, medical or medical information management specialty. A pilot study was conducted with a sample (N = 15). Cronbach's Alpha test indicated that the items were internally consistent (α = 0.723). Questionnaires were distributed by one of the researchers among students of medicine in hospitals and nursing and midwifery students in nursing and midwifery school of Urmia. Data were analyzed using SPSS statistical package (16.0 Version). Results are expressed as Spearman rank correlation coefficient to explore the relationship between patient safety and nominal variables, and one-way analysis of variance (ANOVA) to compare the mean scores of the students’ perceptions, knowledge and attitudes.

## Results

121 out of 134 students (90% return rate) completed the questionnaires. Sixty two (52%), 34 (29%) and 23 (19%) of the students were medical interns, nursing and midwifery students, respectively. Forty-six students were male and 75 were female. Students of nursing and midwifery were on their third or last years of their studies.

Sixty percent of the students agreed with the statement that "medical errors are unavoidable". Eighty percent of the students believed that the provided care was not the best possible care. Sixty four percent of students disagreed with the statement that "health care providers do not make harmful medical errors". Sixty four percent of students agreed with the following statement: "Most errors are due to things that physicians cannot do anything about".

In risk management questions (5–12), more than 50% of students disagreed to report errors after its occurrence and 67% of students disagreed with the statement, "medical errors mean imminent harm to the patient". Seventy three percent of students had negative opinions about "medical error reporting". There was a significant difference between medical, nursing and midwifery students' perceptions towards patient safety (Table 1).

**Table 1:** Student's Perception on Patient Safety (n = 121).

Attitude Items		Students	Strongly Agree	Agree	Neutral	Disagree	Strongly Disagree
Causes of Errors	Making errors in medicine is inevitable.	Medicine	16.1	53.2	9.7	19.4	1.6
		Mid.-Nur.*	20.3	28.8	5.1	30.5	15.3
	There is a gap between what physicians know as “best care” and what is being provided on a day-to-day basis	Medicine	9.7	48.4	22.6	16.1	3.2
		Mid.-Nur.	47.5	45.8	1.7	5.1	0
	Competent physicians do not make medical errors that lead to patient harm	Medicine	0	19.4	16.1	53.2	11.3
		Mid.-Nur.	8.5	15.3	13.6	47.5	15.3
	Most errors are due to things that physicians cannot do anything about	Medicine	16	51.6	14.5	25.8	6.5
		Mid.-Nur.	11.9	64.4	13.6	8.5	1.7
Error Management	If I saw a medical error, I would keep it to myself	Medicine	48	27.4	25.8	40.3	1.6
		Mid.-Nur.	3.4	10.2	22	45.8	18.6
	If there is no harm to a patient, there is no need to address an error	Medicine	16	27.4	8.1	54.8	8.1
		Mid.-Nur.	5.1	15.3	8.5	49.2	22
	Only physicians can determine the causes of a medical error	Medicine	29	33.9	9.7	25.8	1.6
		Mid.-Nur.	6.8	27.1	16.9	39	10.2
	Reporting systems do little to reduce future errors	Medicine	6.6	8.2	18	54.1	13.1
		Mid.-Nur.	10.2	25.4	13.6	30.5	20.3
	After an error occurs, an effective strategy is to work harder and to be more careful	Medicine	33.9	45.2	11.3	8.1	1.6
		Mid.-Nur.	40.7	52.5	6.8	0	0
	Physicians should not tolerate uncertainty in patient care	Medicine	6.5	45.2	33.9	14.5	0
		Mid.-Nur.	8.9	44.6	23.2	17.9	5.4
	The culture of medicine makes it easy for providers to deal constructively with errors	Medicine	4.9	45.9	34.4	14.8	0
		Mid.-Nur.	8.6	39.7	36.2	15.5	0
	Physicians routinely report medical errors	Medicine	3.2	24.2	16.1	45.2	11.3
		Mid.-Nur.	5.1	13.6	11.9	33.9	36.6

*Midwifery - Nursing

In five specific questions about knowledge on patient safety, 40%-50% of the students' self-appraisal of their own knowledge was "good". However, when asked to rate their own knowledge in a general manner, more than 40% of students rated their own knowledge as "poor", and only 20% of them rated their own knowledge as "good". There were some differences among medical, nursing and midwifery students in this regard (Table 2).

**Table 2:** Student's Knowledge on Patient Safety (n = 121).

Knowledge Items*	Students	Very Good	Good	Fair	Poor	Very Poor
The number of preventable adverse events each year in MOHH	Medicine	1.6	17.7	35.5	33.9	11.3
	Mid.-Nur.	6.8	22	18.6	40.7	11.9
The number of preventable adverse events each year reported by international bodies, e.g. IOM Report: To Err is Human	Medicine	5	18.3	36.7	33.3	6.7
	Mid.-Nur.	6.8	20.3	28.8	28.8	15.3
Estimate of the percentage of hospitalizations with adverse events	Medicine	1.6	27.4	27.4	29	14.5
	Mid.-Nur.	6.9	17.2	20.7	37.9	17.2
Characteristics of a successful error reporting system	Medicine	0	22.8	31.6	35.1	10.5
	Mid.-Nur.	3.5	14	21.1	38.6	22.8
Definition of latent factors	Medicine	6.7	10	25	38.3	20
	Mid.-Nur.	5.3	26.3	21.1	29.8	17.5
You are well informed on ‘patient safety’	Medicine	3.2	29	32.3	27.4	8.1
	Mid.-Nur.	6.8	10.2	32.3	45.8	5.1

* MOHH denotes ministry of health hospitals, and IOM Institute of Medicine

About 80% of students required more education on patient safety topics. The findings showed that nursing and midwifery students were more interested in learning patient safety topics than the students of medicine (Table 3).

**Table 3:** Student's Responses to Teaching Items of Patient Safety (n = 121).

Teaching of Patient Safety		Students	Strongly Agree	Agree	Neutral	Disagree	Strongly Disagree
Education	Physicians should routinely spend part of their professional time working to improve patient care	Medicine	19.7	52.5	13.1	13.1	1.6
		Mid.-Nur.	35.6	57.6	3.4	3.4	0
	‘Patient safety’ is an important topic	Medicine	24.6	52.5	16.4	4.9	1.6
		Mid.-Nur.	66.1	27.1	3.4	3.4	0
	Learning how to improve patient safety is an appropriate use of time in medical school	Medicine	14.5	51.6	17.7	11.3	4.8
		Mid.-Nur.	55.9	35.6	5.1	3.4	0
	You would like to receive further teaching on patient safety	Medicine	11.3	45.2	27.4	16.1	0
		Mid.-Nur.	47.5	42.4	6.8	1.7	0
Skills	Supporting and advising a peer who must decide how to respond to an error	Medicine	11.3	50	29	6.5	3.2
		Mid.-Nur.	27.1	50.8	16.9	5.1	0
	Analyzing a case to find the cause of an error	Medicine	11.3	53.2	24.2	6.5	4.8
		Mid.-Nur.	33.9	39	16.9	10.2	0
	Disclosing an error to a patient	Medicine	8.1	29	14.5	30.6	17.7
		Mid.-Nur.	25.4	42.4	11.9	11.9	8.5
	Disclosing an error to a faculty member	Medicine	24.2	51.6	14.5	8.1	1.6
		Mid.-Nur.	45.8	42.4	6.8	3.4	1.7

There were not significant relationships between students' perception of patient safety and their field of study, gender and year of entry to the university. The findings of this study showed that there were significant relationships between students' interest in patient safety education and their gender (p = 0.001), field of study (p = 0.017), and year of entry to university (p = 0.003). Nursing students, males and junior and senior students were more interested in patient safety education. The results of Pearson product moment correlation coefficient showed a significant relationship between students' perceptions of patient safety and their attitudes towards patient safety education (r = 0.344, p = 0.01).

Nursing and midwifery students obtained significantly higher scores on perception towards patient safety than medical students (Table 4).

**Table 4:** Comparison of Mean Squares of Perception on Patient Safety and Attitude to Patient Safety Education Based on Course of Study (n = 121).

		Sum of Squares	df	Mean Square	F	Sig.
Attitude	Between Groups	281.157	2	140.579	7.613	0.001
	Within Groups	2142.019	116	18.466	-	-
	Total	2423.176	118	-	-	-

		Sum of Squares	df	Mean Square	F	Sig.
Knowledge	Between Groups	769.574	2	384.787	19.834	0.000
	Within Groups	2250.392	116	19.4	-	-
	Total	3019.966	118	-	-	-

## Discussion

After the selection of clinical governance as a model of health care quality improvement in 2009, the Iranian Ministry of Health and Medical Education initiated patient-safety-friendly hospitals in 2010 started with a pilot phase in 10 hospitals and expanded to other hospitals. Therefore, patient safety culture is a relatively new field in this country [21]. Medical and paramedical staff are the most important components of patient safety [18]. On the other hand, to think strategically and considering long term objectives, focusing on attitudes, preferences and knowledge of medical and paramedical students is necessary for a successful plan for patient safety in the future and making cultural changes sustainable [9, 11].

This study showed some important points regarding patient safety education. The noticeable findings of the study were: inadequate knowledge on patient safety, a relative difference between students of medicine and nursing/midwifery students, and the necessity of patient safety education.

Regarding the causes of medical errors, the majority of respondents claimed that medical errors are inevitable, human factors play an important role; and failure in providing the best care for patients. This represents a basic misconception about the nature and type of human error. The results regarding the causes of errors and quality of care were in line with the studies of Leung [20] and Yoshikawa [22], as well as Abdi’s, except the result on the inevitability of errors [23].

In the field of medical error management as a subdivision of perception, the following factors were taken into consideration: Error-reporting system, near miss, determining the causes of errors, use of effective strategy, acceptance of uncertainty and cultural factors of medicine. In this subdivision, the first five items were in line with Leung study [20]. Regarding error management, the results related to error reporting, near miss, determining the causes of errors items were in line with Abdi’s investigation while the error reporting system and culture of medicine items were not compatible with his study [23].

In the category of error management, the results were similar to the findings of Yoshikawa. Regarding patient safety, nursing/midwifery students have deeper perceptions comparing to the students of medicine. This can be explained by the emphasis which was given by the faculty of nursing/midwifery to the subject of patient safety training. According to the Yoshikawa, there was no significant difference between the perceptions of nursing/midwifery and students of medicine regarding patient safety [22]. Kohestani reported an error rate report of 75% by nursing students [24]. In this study the error rate’s report by students of nursing/midwifery, and medicine were 64 and 42%, respectively.

Concerning the level of knowledge about patient safety, the students of nursing and midwifery dedicated more knowledge on patient safety compared with the students of medicine. Leung’s study appeared to have the same duality in patient safety [20]. The results of this study are consistent with those of Abdi in the form of direct and detailed questions [23].

In this study, 25% of nursing-midwifery students and in the survey of Khalili, half of respondents were aware of relevant state authorities which formulate and manage patient safety activities across the country [25]. In this study also, knowledge level of nursing/midwifery students was higher than that of the students of medicine which is inconsistent with Kerfoot study. In Kerfoot study, patient safety knowledge varied by the degree and discipline of the students [26].

The respondent’s attitudes toward patient safety education were positive. In his study, Leung indicated that the majority of respondents emphasized the necessity of patient safety education, necessary training skills and time allocation in the related courses [20]. Bowman has reported the necessity of student's desire to teaching patient safety [27]. Patey has reported lack of adequate knowledge of the students on patient safety [28]. Abdi also has reported low knowledge and negative attitude of the students to patient safety in pre-intervention phase [23].

A group of students (33%) did not accept the inevitability of errors and about one quarter of them stated that the expert doctors never make errors, which reflects a misunderstanding of the nature and pattern of human errors [20]. Failure to report of near miss errors indicates unawareness of its consequences and potential effects on the improvement of services. Using effective strategy after error occurrence indicates lack of understanding of the complexity of the system and the processes of treatment and care. One third of respondents were neutral about medicine culture that reflects their confusion and lack of proper induction of the safe care, error management and statutory formal training of patient safety.

## Conclusion

Medical and paramedical students in Iran were familiar with medical errors as an unavoidable barrier between ‘best care’ and what is actually provided. However, there was little knowledge about the multi-factorial mechanisms underlying occurrence of errors. Also, a knowledge gap and interest to learn patient safety was found. Establishing formal curriculum on patient safety and maintaining this change in health care culture is essential. Also, it is recommended to formulate better policies for patient safety. These will finally reduce all types of errors in complex environments such as hospitals and will make quality improvement in clinical practice.
